# Role of Interventional Radiology in the Management of Peripheral Vascular Malformations: A Tertiary Care Center Experience

**DOI:** 10.7759/cureus.2335

**Published:** 2018-03-16

**Authors:** Misbah Tahir, Muhammad Anees Mumtaz, Anum Sultan, Jawaid Iqbal, Raza Sayani

**Affiliations:** 1 Radiology, Liaquat National Hospital and Medical College; 2 Medical College, Liaquat National Hospital and Medical College; 3 Department of Radiology, The Aga Khan University, Karachi.

**Keywords:** sclerotherapy, vascular malformation, sodium tetradecyl sulphate, bleomycin, ethanol, ethanolamine oleate, glue, covered stents, coils, polyvinyl alcohol particles

## Abstract

Peripheral vascular malformations (PVMs) represent a wide spectrum of vascular abnormalities occurring due to anomalous connections between arteries, veins, capillaries, and lymphatic channels at the microscopic level, in different combinations. They are rare and challenging to treat. Different operators may have different approaches based on their experience and expertise. Sclerotherapy either alone or in combination with embolization has been used as an independent method for the treatment of PVMs.

Purpose

The aim of this study is to assess the safety and efficacy of sclerotherapy and embolization, with or without surgery, for the treatment of peripheral vascular malformations, based on our approach.

Materials and methods

A retrospective review of all patients with PVMs treated in our interventional radiology department from 2011 to 2017 was carried out. Medical records, imaging, and follow-up notes were reviewed to evaluate the response to treatment and post-procedure complications.

Results

Thirty-four sessions were performed in 15 patients (eight male, seven female) with PVMs. Low-flow lesions were identified in 10, intermediate flow in one, and high flow in four patients. Sodium tetradecyl sulfate (STS) was used as the sclerotherapeutic agent in 10 (66.67%), glue with lipoidal in three (20.0%), and bleomycin in one patient (6.67%). Coils with PVA and a covered stent were used in one and a combination of coil, PVA, and gel foam was used in one patient. A marked response was seen in 11 and a partial response in four patients. One patient developed foot gangrene. Stent thrombosis was noted in one patient with no clinical consequences. Recurrence was seen in two patients, who were lost to follow up.

Conclusion

PVMs are complex lesions. Sclerotherapy with or without embolization is a safe and effective treatment modality, with clinical response approaching 100%.

## Introduction

Peripheral vascular malformations (PVMs) represent a wide spectrum of vascular abnormalities occurring due to anomalous connections between arteries, veins, capillaries, and lymphatic channels at the microscopic level, in different combinations [1]. These lesions are characterized as low-, intermediate-, and high-flow lesions depending upon the flow velocity. Symptoms such as pain, swelling, functional disabilities, and cosmetic deformity can lead to significant morbidity.

Sclerotherapy either alone or in combination with embolization has been used as an independent method for the treatment of PVMs. It can also be used as an adjunct to surgery [2-3]. Various agents are used in sclerotherapy and the embolization of PVMs, including sodium tetradecyl sulfate (STS), bleomycin, ethanol, ethanolamine oleate, glue, covered stents, coils, polyvinyl alcohol particles (PVA), and gel foam. The selection of sclerotherapeutic and embolizing agents vary depending on the location and characteristics of vascular malformations and radiologist preference. As PVMs are rare in occurrence, challenging to treat, and there is significant variation in the choice of sclerotherapeutic and embolizing agents among different operators depending on their personal preference and experience, we wanted to see the safety and efficacy of our initial experience in treating PVMs.

## Materials and methods

In this study, we carried out a retrospective review of all peripheral vascular malformations (PVMs) treated in our vascular interventional radiology (VIR) department from 2011 to 2017. Informed consent was obtained from all patients. The diagnosis of PVMs was made on the basis of physical examinations and imaging. Imaging modalities included ultrasound (U/S) and magnetic resonance imaging (MRI) in all patients. In high- and intermediate-flow lesions, angiography was also performed. U/S imaging was done on Toshiba Xario 100 (Canon Medical Systems Corporation, Tochigi Prefecture, Japan) using 5 MHz and 7.5 MHz high-frequency probes with color Doppler. Magnetic resonance imaging (MRI) was done on Toshiba Excelart Vantage (ATLAS) 1.5 Tesla (Canon Medical Systems Corporation, Tochigi Prefecture, Japan). The MRI protocol included T1-, T2-weighted images with and without fat saturation and post-contrast T1-weighted images with fat saturation in the axial, coronal, and sagittal planes.

Lesions were characterized as high, intermediate, and low flow depending on their flow pattern and velocities. Lesions showing only monophasic venous flow were labeled as low flow and those with a predominant arterial flow pattern were labeled as high flow. PVMs with predominant venous low but also having arterial flow were taken as intermediate flow. Sclerotherapy and embolization were performed under real-time U/S and fluoroscopic guidance using STS (3% sodium tetradecyl sulphate, Setrol, Samrath Life Sciences, Mumbai, India), glue (Glubran, GEM S.R.L, Viareggio, Italy) with lipoidol (lipoidol ultra-fluid 480 mg/ml, Guerbet, Istanbul), bleomycin (Bleomycin 15mg, Fresenius Kabi, USA), covered stent (wall graft, Boston Scientific, USA), gelfoam (Equispon, Eguimedical BV, The Netherlands), coils (2D Helical-35, Boston Scientific, Cork, Ireland), and PVA particles (Contour, Boston Scientific, USA). Sclerotherapeutic and embolizing agents were selected according to the location and characteristics of PVMs.

STS was used for low and intermediate-flow lesions and high-flow lesions were embolized using a covered stent, coils, PVA, gel foam, glue, and bleomycin in various combinations, as shown in Table 1.

**Table 1:** Patients' information according to region involved, presenting symptoms, flow characteristics, number of sessions, agents used, response, and follow-up.

Patient	Age (years)	Gender	Location of PVMs	Presenting symptoms	Flow	No. of sessions	Sclerosing agent	Clinical response	Outcome	Complications	Duration of follow-up
1.	10	Male	Bilateral parapharyngeal spaces	Progressive dyspnea and dysphagia. Dyspnea aggravated during sleep, resulting in an inability to go into deep sleep.	Low	3	STS	Resolution of dyspnea	Marked response	None	19 months
2.	16	Female	Planter and dorsal aspect of right foot	K/c right foot vascular malformation, pain in right foot	Low	1	STS	Resolution of pain	Marked response	Distal foot gangrene	16 months
3.	37	Female	Right parapharyngeal space extending into larynx	Painless swelling in the neck on the right side for last 2 yrs that is progressively increasing in size	Low	2	STS	50% reduction in swelling	Partial response	None	16 months
4.	23	Female	Posterior aspect of left calf	Pain in the left leg for 10 yrs that was increasing in severity for the last 2 yrs	Low	1	STS	Resolution of pain	Marked response	None	14 months
5.	31	Male	Lateral aspect of right elbow	Severe pain in right arm	Low	3	STS	Resolution of pain.	Marked response	None	4 yrs
6.	35	Male	Left eyelid	Ptosis of left eyelid, difficulty in opening the eye, no visual impairment	High	1	Bleomycin	Significant resolution. He was able to open his eye.	Partial response	None	Lost to F/U.
7.	32	Male	Right ankle	Pain in right ankle extending into calf	Low	7	Glue with lipiodol	50% reduction in size on post-procedure MRI	Partial response	None	5 yrs
8.	18	Female	Right lower limb	Multiple lesions in right lower limb, pain in right lower limb	Low	2	STS	Resolution of pain	Marked initial response, recurrence on 2-yr F/U	None	3 yrs
9.	18	Female	Left arm	Left arm pain and swelling	High	2	Glue with lipiodol, wall-graft stent.	Resolution of pain and swelling	Marked response	None	4 yrs
10.	22	Female	Right distal thigh	Pain and swelling in right distal thigh since childhood increasing for 2-3 yrs	Low	3	STS	Resolution of pain	Partial response, recurrence on 3yr F/U	None	4 yrs
11.	20	Male	Face	Bleeding from gums	High	1	Coils with PVA and gel foam	Cessation of bleeding	Marked response	None	4 yrs
12.	17	Male	Right arm	Pain on compression, swelling increasing in size	Low	2	STS	Resolution of pain and swelling	Marked response	None	3 yrs
13.	18	Female	Face	Extensive vascular malformation on face	High	2	Coils with PVA	Complete resolution	Marked response	None	4 yrs
14.	22	Male	Left forearm	Pain and swelling increasing in size for last 3 months	Intermediate	1	STS, Glue with lipiodol	Resolution of pain and swelling	Marked response	None	13 months
15	22	Male	Neck	Diffuse neck swelling with difficulty in breathing and swallowing	low	2	STS	60% to 70% resolution of swelling and symptoms	Marked response	None	5 months

After localizing the lesion under U/S, cannulation with a needle (20G, 22G, 25G BD spinal needle, Becton Dickinson SA, Spain) was done and contrast (Iomeprol, Braccos PA, Milano, Italy) was injected to delineate the lesion on fluoroscopy. A percutaneous injection of sclerotherapeutic and embolizing agents was done using U/S and fluoroscopic guidance. The outflow veins were occluded or compressed using a tourniquet during the injection, wherever possible. STS foam was created with STS mixed with air and contrast, each in equal amounts. People have mixed STS with lipiodol but because of the financial constraints of many patients in Pakistan, we are using non-ionic contrast instead of lipiodol. Glue was usually injected in combination with lipiodol in a 1:1 ratio. The amount of glue and lipiodol varied according to the lesion size and response. The injection was stopped when thrombosis of vascular channels was seen on U/S and a loss of flow was identified on angiography. However, angiography was done only in selected cases in intermediate- and high-flow lesions.

Technical success is defined as the optimal injection of sclerosant or the exclusion of lesion by embolization as demonstrated by angiography or U/S. Clinical success is assessed by a reduction in pain and an improvement in swelling and function. Clinical response is categorized as marked response, partial response, and no response. Patients showing complete resolution of symptoms were labeled as having marked response, those showing a reduction in symptoms as partial response, and as no response when no change in symptoms was noted. Clinical outcomes were determined by reviewing all the pre- and post-procedure imaging and follow-up notes in the interventional radiology clinic. MRI was not routinely done in the follow-up of all patients. Follow-up of all patients included clinical evaluation, physical examination, and grayscale and color Doppler U/S.

## Results

Thirty-four sessions were performed in 15 patients (eight male, seven female) with PVMs. The mean age of patients was 22.7 years (range 10-37 years). Nine patients had pain (60.0%), six had a swelling (40.0%), one had ptosis (6.67%), two had dyspnea affecting sleep significantly (13.34%), and one patient presented with life-threatening oral hemorrhage (6.67%). One patient with low-flow vascular malformation in a lower limb had prior surgery with no significant response. The average follow-up duration was 2.71 years (0.4-5 years). The distribution of vascular malformation according to flow characteristics and region of involvement is shown in Table 2.

**Table 2:** Distribution of vascular malformations according to flow characteristics and region of involvement

Region involved	PVMs flow characteristics			Total
	High flow	Intermediate flow	Low flow	
Upper limb	1	1	2	4
Lower limb	0	0	5	5
Head and neck	3	0	3	6
Total	4	1	10	15

STS alone was used as the sclerotherapeutic agent in nine patients. Glue with lipiodol was used in three patients. Out of these, one had covered stent placement and another had STS injection. Bleomycin was used in one patient. Coils along with PVA particles were used in one patient and a combination of coils, PVA, and gel foam was used in one patient with a high-flow vascular malformation. A 100% technical success rate was achieved. Clinical success was 100%. A marked clinical response was seen in 11 out of 15 patients (73.34%) with a resolution of symptoms. Four out of 15 (26.67%) patients showed partial response and no patients were noted in the no response category. One patient developed complication in the form of gangrene in the toes and the second to fifth toes were amputated. Stent thrombosis was noted in one patient without any clinical consequences or functional restriction. Therefore, we did not go for further intervention for the restoration of stent patency. A recurrence was seen in three patients who were lost to follow-up and visited us two and three years later.

## Discussion

PVMs are rare and challenging lesions to treat with an estimated prevalence of 1.5 % in the population [4]. A multidisciplinary approach, including an interventional radiologist, surgeon, dermatologist, hematologist, pathologist, and oncologist, is critical in the diagnosis and management of PVMs. Many times, surgical excision because of the proximity of vital structures is not possible for the treatment of PVMs. Therefore, it is of immense importance that the interventional radiologist is aware of the available treatment techniques and is skilled in their implementation, keeping in view the associated benefits, complications, and outcomes.

In our study, we have performed sclerotherapy in two patients having vascular malformations in the foot. One patient had a vascular malformation in both the planter and dorsal aspects of the foot. Surgery was attempted but complete excision was not achieved. At three different sites, STS foam admixed with glue and air was injected and a compression bandage was applied. The patient developed distal foot gangrene and all toes were amputated. There was a complete response with a resolution of pain and lesion on follow-up. In our opinion, this complication developed due to the tight compression bandage applied after the procedure and the inability of duty doctors to appreciate an ischemic event in a timely manner. Moreover, this patient also had a previous surgery of the foot that led to compromised vascular supply and may have contributed to the development of gangrene. Therefore, care should be taken in applying a compression bandage after sclerotherapy so that a venous outflow obstruction resulting in the swelling of the limb or vascular compromise should not occur. Close patient observation is necessary following the sclerotherapy of lesions involving limbs distally. Weiss et al. reported that 30 mmHg support stockings worn for three weeks after sclerotherapy is not only effective immediately after injection but also yield better results in the long term [5]. The two other patients had a vascular malformation in the lower limb, one in the foot and the other in the thigh. Sclerotherapy was done using STS in both patients. There was a complete clinical response with no complication; however, they were lost to follow-up and later presented with a recurrence of the disease and symptoms. This signifies the fact that long-term follow-up is mandatory in PVMs for achieving marked clinical success. Patients who are lost to follow-up may present with a recurrence of symptoms.

**Figure 1:**
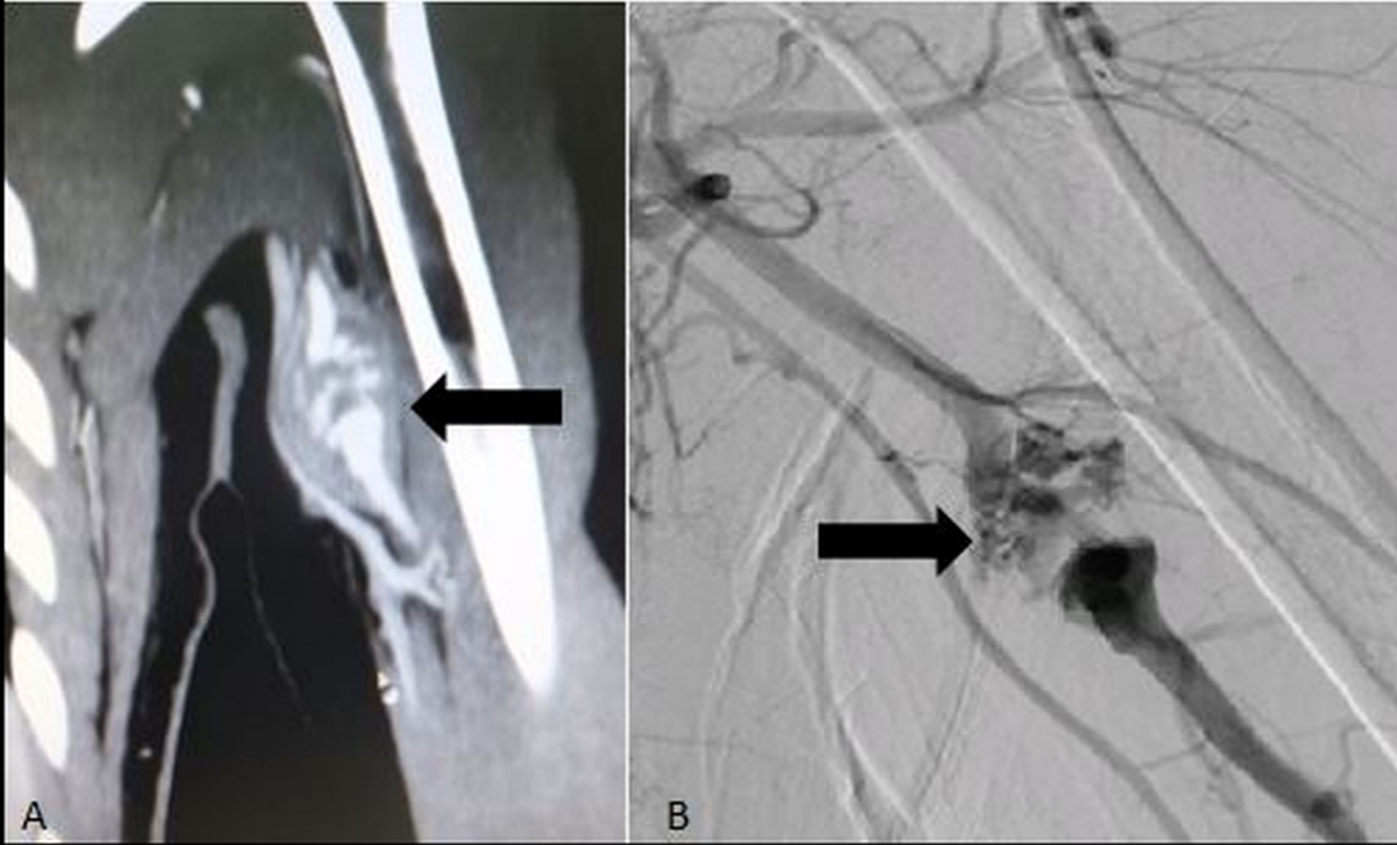
CT scan with contrast (A) and digital subtraction angiogram (B) of an 18-year-old patient showing multiple tiny outpouchings (Arrows) arising from the brachial artery

An 18-year-old female presented with pain in the left upper limb. Imaging revealed multiple, tiny outpouchings arising from the brachial artery, which were excluded by placing a covered stent (Figure 1).

**Figure 2:**
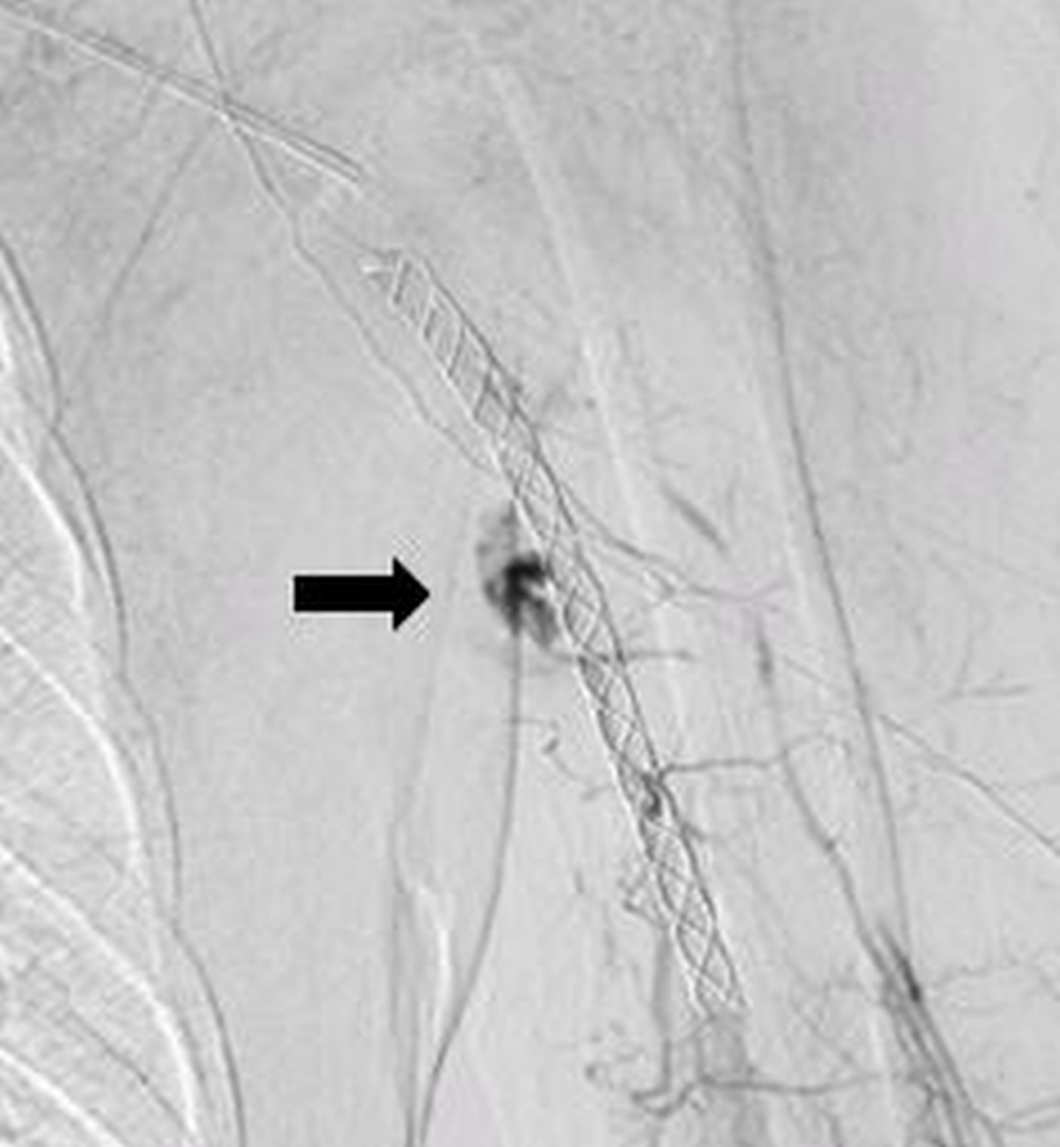
Post-stenting angiogram showing a few vascular channels (Arrow) filling in the venous phase

Post-stenting angiogram revealed a few vascular channels filling in the venous phase (Figure 2). 

**Figure 3:**
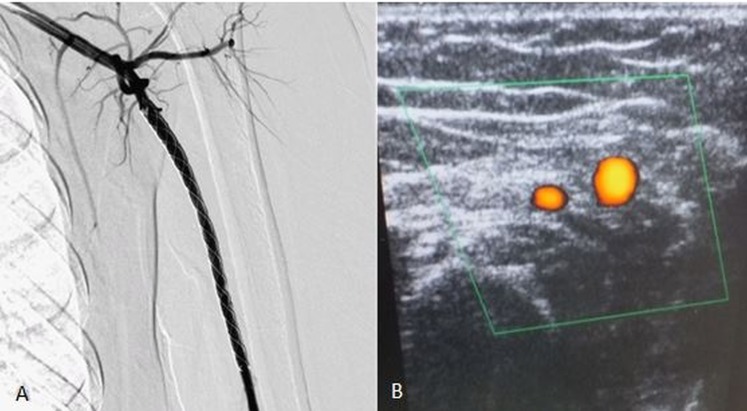
Post-procedure angiogram (A) and Doppler ultrasound (B) showing the complete exclusion of the lesion

These were embolized with a percutaneous injection of glue, resulting in the complete exclusion of the lesion (Figure 3). 

At the three months follow-up, the occlusion of the stent was noted with the development of collaterals from the profunda brachii artery, re-forming the brachial artery with a good distal flow. There was no pain or limitation of movement, so no further intervention was done and the patient has been asymptomatic for the last three years. We did not include it as a complication. Out of 15 patients, nine have vascular malformations in the limbs and only one patient developed complications in the form of gangrene. Ali S et al. reported a 20% complication rate in the treatment of venous malformations treated with percutaneous sclerotherapy [6]. Our experience shows a low risk of complication (6.67 %) in patients with PVMs. It is significantly lower as compared to the previous studies [7-9].

Castren E et al. [10] published a series of 75 patients who underwent sclerotherapy for vascular malformations (VM) in the region of the head and neck with a 17.3% complication rate. In our study, we treated six patients with vascular malformations in the region of the head and neck with a significant improvement in pain, swelling, and function without any complication. One patient had extensive vascular malformation involving the carotid, pharyngeal, and parapharyngeal spaces, causing a significant compression of the airway, resulting in an inability to sleep. To avoid the compression of the airway from inflammation and edema, a tracheostomy was done before the sclerotherapy. His follow-up MRI after two sessions of sclerotherapy revealed intermediate and low signals appearing in a previously noted homogenously T2W hyperintense lesion. A concomitant laryngoscope examination revealed a decrease in the size and pressure effect of VM over the airway. A marked clinical response was noted in this patient with significant improvement in his symptoms. In another patient, a high-flow vascular malformation was noted in the upper eyelid, causing marked ptosis of the eyelid. Sclerotherapy with bleomycin was performed and partial response was noted after the procedure. This patient didn’t turn up for follow-up.

**Figure 4:**
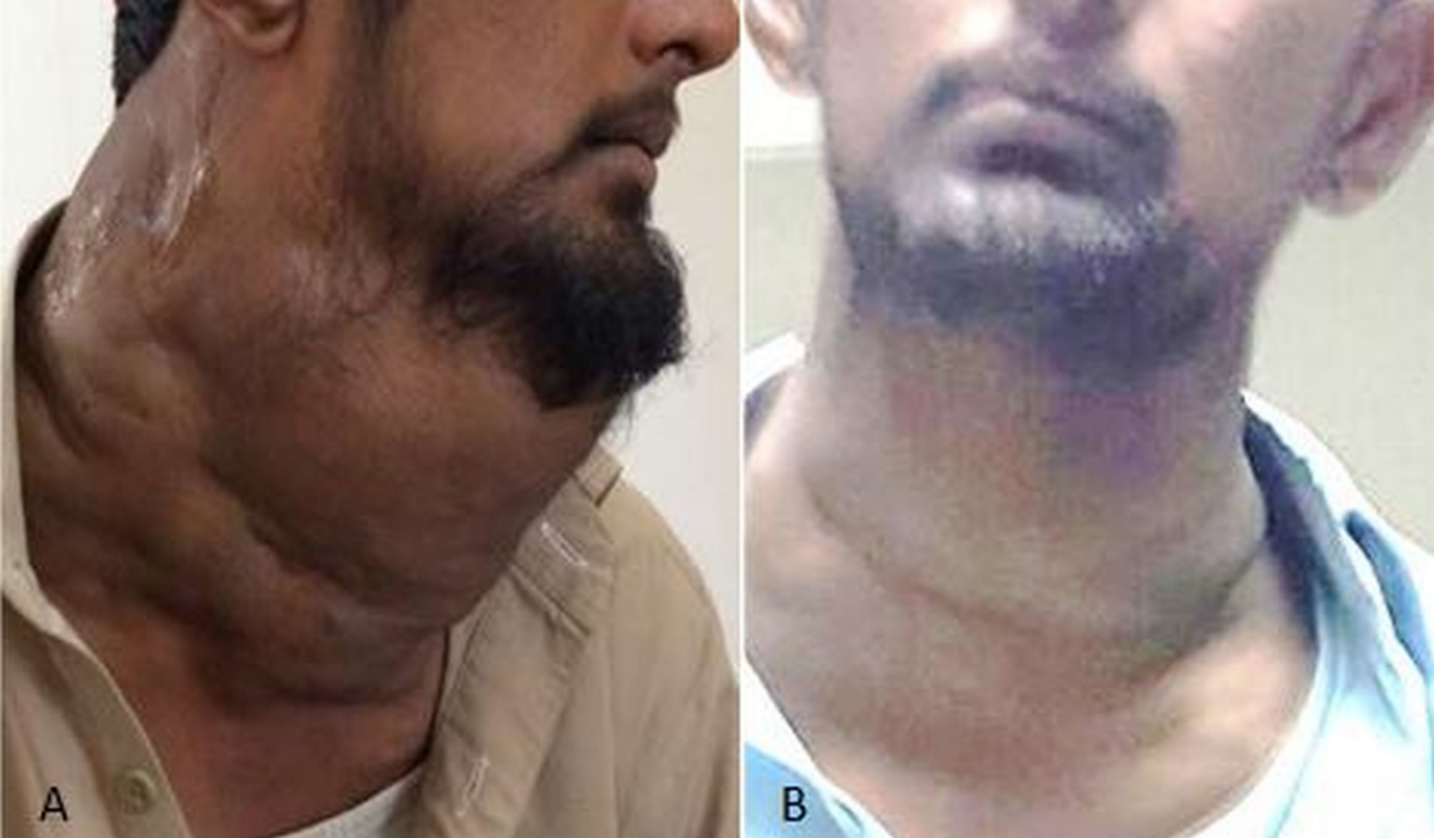
A. Patient presented with a diffuse neck swelling. B. Marked reduction noted in the neck swelling after sclerotherapy with STS STS: sodium tetradecyl sulfate

A 22-year-old male presented with a huge neck swelling, which caused episodic dyspnea. A computed tomography (CT) scan revealed significant pressure over the airway. Two sessions of sclerotherapy were performed after endotracheal intubation. A significant reduction in swelling with a resolution of dyspnea was noted in the five-month follow-up (Figure 4).

None of our patients with a vascular malformation in the head and neck developed complications after the procedure.

In the literature, there is a difference in the choice of sclerotherapeutic agents among different authors. We have used STS as the sclerotherapeutic agent in most of our procedures, as it is one of the most-effective and safest agents used for sclerotherapy, with a success rate comparable to ethanol [11]. However, it can cause various adverse reactions, such as sloughing and necrosis of tissues, skin discoloration, and allergic reactions. The incidence of allergic reactions after STS injections ranges from 0.15% to 0.30%. In our study, only one patient developed an allergic response to the test dose of STS. This patient had seven sessions of sclerotherapy. Bleomycin was also given but there was no clinical response. In this patient, we used glue admixed with lipiodol for sclerotherapy. He showed a marked clinical response. Allergic and anaphylactic responses were also reported with the use of STS in previous studies. In our study, wherever possible, we used glue with lipiodol in high- and intermediate-flow lesions and STS in low-flow lesions, and we found it a very effective combination.

The majority of patients with PVMs may require multiple sessions. In our study, only five out of 15 patients (33.3%) had a single session. Six out of 15 patients (40%) had two sessions. Three out of 15 patients (20%) had three sessions and one patient had seven sessions.

The results of our study demonstrated a marked response in 73.34% of patients and a partial response in 26.67% with a 0% failure rate.Two patients with high-flow malformation underwent surgery after sclerotherapy with a complete excision of the lesion. With the mean follow-up of 2.71 years, we conclude that sclerotherapy with or without embolization is a safe and effective treatment modality, with most patients experiencing symptom relief. STS is a safe and effective treatment modality; however, it needs multiple sessions and long-term follow-up. Special attention, with effective monitoring, should be done for distal extremity and head and neck lesions.

The limitations of our study are that it is a retrospective study with a small sample size.

## Conclusions

PVMs are complex lesions. Sclerotherapy, with or without embolization, is a safe and effective treatment modality with clinical success approaching 100%. Many patients will need multiple sessions, and long-term follow-up is the key to success.
